# Development of a *Plasmodium vivax* malaria vaccine for clinical applications using transgenic parasites, virus-like particles and recombinant viruses

**DOI:** 10.1186/1475-2875-13-S1-P77

**Published:** 2014-09-22

**Authors:** Arturo Reyes-Sandoval, Shahid Khan, Chris Janse, Martin Bachmann, Adrian Hill

**Affiliations:** 1 University of Oxford, Oxford, UK; 2 Leiden University Medical Center, Leiden, The Netherlands

## 

As plans for malaria eradication continue, a vaccine targeting *Plasmodium vivax* is of major importance due to the fact that it is the most widely distributed human malaria parasite in the world and the most difficult to eliminate due to its ability to hide in the liver forming hypnozoites. Malaria liver-stage vaccines are one of the leading strategies and the only approach that has demonstrated complete, sterile protection in clinical trials. We have developed vaccine candidates using platforms suitable for human use, consisting of virus-like particles (VLPs), chimpanzee adenovirus (ChAd63) and modified vaccinia Ankara (MVA) containing malaria pre-erythrocytic antigens, and several novel challenge models using transgenic mouse-malaria *P. berghei* parasites expressing *P. vivax* antigens that permit the assessment of vaccine efficacy in small animal models, thus providing a suitable platform for vaccine development in non-endemic countries and permitting the progress of vaccines that rely on assessment of efficacy rather than measurement of immunogenicity, in the absence of a conclusive correlate of protection.

We present results of two leading malaria vaccine candidates, a universal *vivax* circumsporozoite antigen (PvCSP) and the thrombospondin-related adhesion protein (PvTRAP) to create recombinant adenovirus, MVA and a platform consisting of VLPs to enhance efficacy of our malaria vaccines. All have been tested using transgenic parasites expressing the *P. vivax* CSP VK210, VK247 and TRAP, thus permitting the assessment of protective efficacy in various mouse strains and yielding important information to address cross protection against CSP using a challenge with VK210 or VK247 parasites in mice vaccinated with homologous, heterologous or chimeric CSP antigens.

Our studies indicate that our vaccines are able to induce outstanding T-cell responses and antibody titers that can protect against a stringent challenge with high doses of fully virulent transgenic parasites in mice. Our groups are currently committing a substantial effort in developing further transgenic parasites to allow the discovery of new vaccine candidates able to afford protection.

